# Moving Toward Universalism? The Future of Long‐Term Care in the United States

**DOI:** 10.1111/1468-0009.70096

**Published:** 2026-08-17

**Authors:** PAMELA HERD

**Affiliations:** ^1^ University of Michigan

**Keywords:** long‐term care, administrative burden, universalism

## Abstract

**Policy Points**

- America's long‐term care system is largely poverty‐based, requiring most older adults to spend down their assets to qualify for limited, Medicaid‐funded support, which is biased toward institutional care and plagued by bureaucratic complexity and enrollment hurdles.
- There are now, however, some promising changes. First, though access to home care still is more difficult than access to institutional care, the use of Medicaid home care services has grown significantly since the 1980s.
- Most promising is Washington State's innovative new universal social long‐term care insurance program. Broad‐based, compulsory social insurance has proved effective in other countries—lessening poverty‐induced eligibility hurdles, increasing access to care (especially in communities), and mitigating political vulnerabilities associated with targeted policies.

**Context:**

America's long‐term care policy approach is poverty‐based, requiring most older adults to spend down their assets to qualify for limited, Medicaid‐funded support, which is biased toward institutional care and plagued by bureaucratic complexity and enrollment hurdles.

**Methods:**

I conduct a review of long‐term care policy developments since the 1980s, as well as related research on the impacts of these varying policy approaches on access to services.

**Findings:**

While Medicaid‐funding long‐term care remains plagued by complexity and access issues, there have been promising changes. Use of home and community‐based services has increased substantially for Medicaid beneficiaries since the early 1980s due in large part to activism on the part of disability rights movement. Though new and still relatively limited in scope, the most promising change has been the recent adoption of a universal long‐term care policy in Washington State.

**Conclusions:**

As the population ages and care needs grow more urgent, the United States faces a critical choice—cling to a piecemeal, confusing, and impoverishing long‐term care system or embrace universalism to ensure meaningful access to needed community‐based long‐term care supports.

Most older adults will need long‐term care support as they age. ^1^ Most Americans, however, not only assume they are unlikely to need that care, but also believe that Medicare pays for it and they will be able to remain in their own home to receive it. The reality is quite different.

Nearly 80% of people who reach the age of 65 will require long‐term care support, Medicare doesn't cover those costs, and most Americans cannot afford to pay for it, with average annual costs reaching around $120,000.^1^, ^2^ Unlike Social Security and Medicare, which are universal in nature, providing income support and health insurance to nearly all older adults, the United States has a “poverty based” long‐term care program, funded mostly by means‐tested Medicaid.^1^, ^3^ Because poverty is an eligibility criterion, most people need to become poor to qualify. Consequently, many middle‐class Americans “spend down” their assets to receive desperately needed care.^4^ Even then, Medicaid is typically only required to cover institutional care. In many states Americans face long wait lists, and an often‐impenetrable bureaucratic maze, to access care in the community.^5^ The situation is likely to worsen in coming years as states grapple with nearly $1 trillion in Medicaid spending cuts from the 2025 One Big Beautiful Bill Act, and as the Trump administration rolls back rules meant to protect people receiving long‐term care.

While this paints a grim picture, there are some remarkable bright spots emerging in long‐term care policy. Innovations at the state level have the potential to fundamentally remake the system. Whereas in the 1980s most Americans were forced into institutions to receive Medicaid‐funded long‐term care, many states have redesigned their systems so that the majority of Medicaid beneficiaries receive care at home. And even more promising, in Washington state there is a move toward universal long‐term care insurance—and a move away from the United States’ convoluted poverty‐based system.

## The Need for Long‐Term Care

Over 80% of Americans who reach age 65 will eventually need long‐term care support.^1^ The kind of support people need varies, from help with basic activities of daily living, such as eating, bathing, and dressing, to instrumental activities of daily living, such as grocery shopping or cooking. The intensity of what people need assistance with increases as people age. For example, while less than 10% of people need assistance with more basic needs, like getting dressed, between the ages of 65‐74, this increases to almost 30% for those between the ages of 85‐94 and more than 60% for those older than 90.^6^ The need for help with things like grocery shopping or cooking has a much higher prevalence—as much as 70% higher.^7^ Although there is evidence that these limitations are declining in more recent older cohorts, population aging means that overall need for long‐term care will remain high.^8^

Notably, however, the intensity of the need varies across groups. For example, cognitive problems tend to elicit the most significant long‐term care costs. And while current projections indicate that those with college degrees at age 65 can anticipate spending 85% of their remaining years in good cognitive health, estimates are that those with less than a high school degree can anticipate just under 50% of their remaining years to be spent in good cognitive health.^9^ More generally, women are projected to spend twice as many years disabled as compared to men, largely due to their increased life expectancy. Black older adults are projected to spend two more years of their lives disabled compared to White older adults, even as they live shorter lives. And those without a high school degree, compared to those with one, will also face that penalty.^10^

## What's Wrong With Our System?

The United States’ long‐term care system has significant problems, which undermine Americans’ ability to receive the kind of care that they say they want. In short, most Americans want to stay in their own homes and receive help without needing to go bankrupt to get it.

### Long‐Term Care Is Expensive

**Figure 1:**
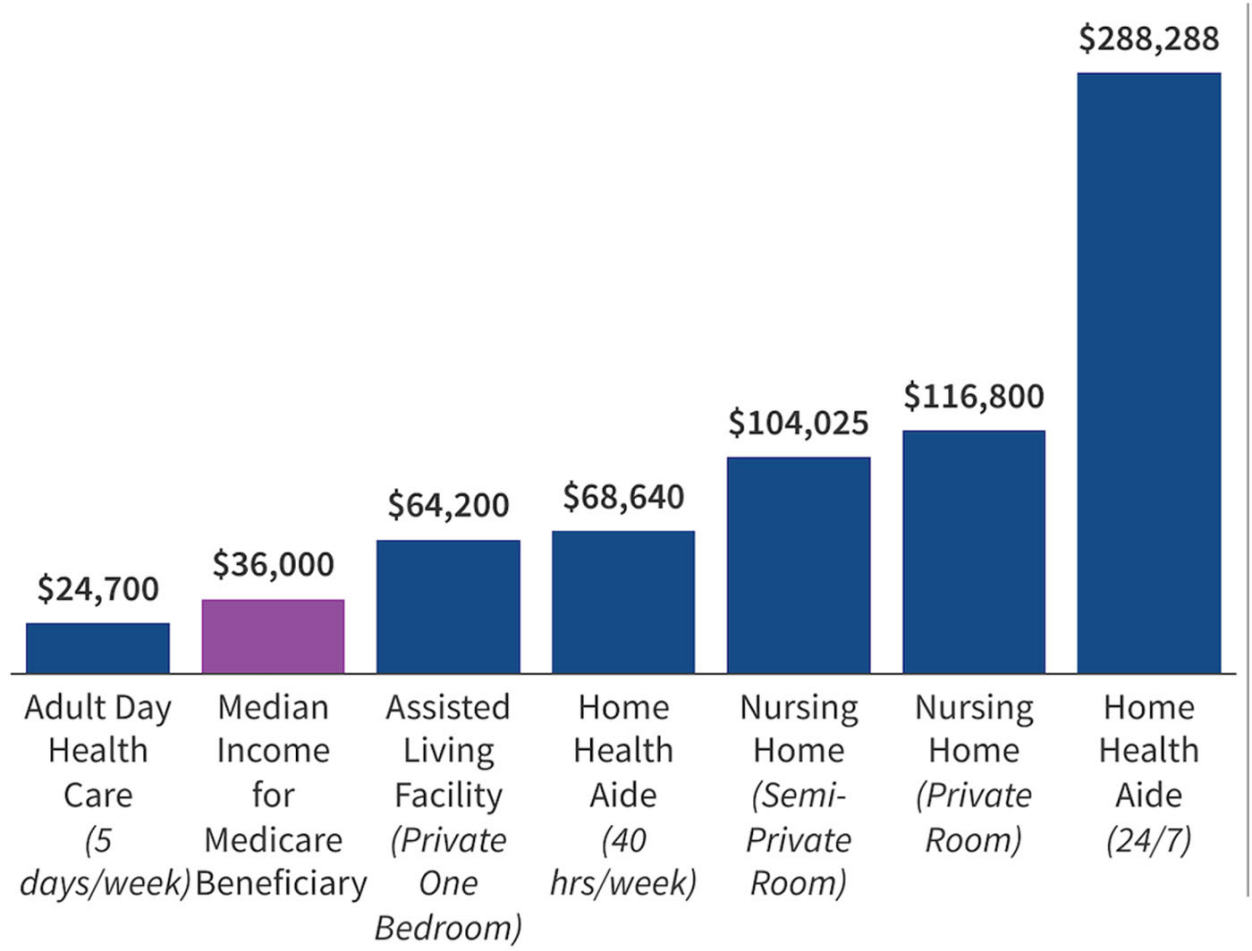
Annual Costs for Long‐Term Care Services in the United States as Compared to the Median Income for a Medicare Beneficiary [Colour figure can be viewed at wileyonlinelibrary.com] *Source*: Reproduced from Chidambaram and Burns 2024.^11^

While those with fewer resources are disproportionately more likely to need long‐term care support, the reality is that very few Americans can afford to pay for it. Figure 1 provides an overview of annual costs for long‐term care, which range from $24,700 for adult day health care to $288,288 for 24/7 home health care.^11^ For a relative perspective, the median monthly income for those aged 65 and older is around $4,700 a month, and for women living alone and minority households, typically half that amount.^12^, ^13^

### A Poverty‐Based Long‐Term Care System

So how do Americans pay for their long‐term care? Notably, although private health and dental insurance is common, private long‐term care insurance is not. In the 1990s, it was touted to solve the long‐term care financing puzzle, but it covers just 8.7% of costs. Varying factors explain its limited role, including the industry underestimating eventual care costs.^14^ Today, many insurers have dropped out of the market, and many individuals who have policies are finding that their coverage isn't as generous as they expected. Because the private market has been so ineffective, even with federal and state subsidies designed to facilitate it, Medicaid remains the principal policy support for long‐term care.

Medicaid is a means‐tested program. Only those with low incomes and limited assets qualify.^15^ In 2026, that means having an income just under $3,000 a month and assets that don't exceed $9,950 for a single adult and $14,910 for a married individual. However, there is a significant amount of variation across states in terms of how assets are calculated in practice.^15^ For example, if only one spouse needs long‐term care, there is variance on which assets are disregarded in the eligibility calculation.

Some have described our long‐term care system as “poverty based” because the reliance on Medicaid actually pushes many into poverty. Middle‐class and even many upper middle‐class older adults don't have the economic resources needed to cover long‐term care costs for extended periods of time. Consequently, they “spend down” their assets to qualify for care.^16^ This has implications not only for the care recipient but also for their spouse or family members. One recent study found that a spouse of Medicaid long‐term care recipient can expect to spend 19% of their income in out‐of‐pocket care costs. Further, they are 8 percentage points more likely to end up qualifying for Medicaid themselves given their household income loss.^17^ Although spouses can keep their home, they must effectively spend down most of their remaining assets, pushing many into poverty themselves.

### Medicaid Is Biased Toward Institutional Care

Almost no one wants to end up in a nursing home. A survey of adults aged 50 and older found that just 1% of individuals prefer institutional care if they eventually need help with basic tasks of daily living.^18^ Nonetheless, the United States’ long‐term care system has an “institutional bias.” In short, the system was designed to subsidize and support people receiving care in long‐term care institutions rather than in their own homes and communities.

**Figure 2:**
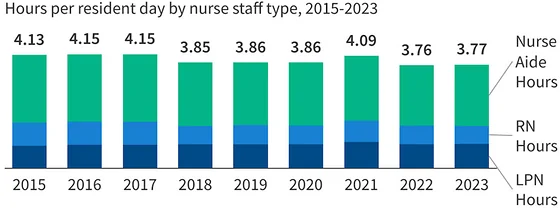
Hours per Resident Day by Staffing Type, 2015‐2023 [Colour figure can be viewed at wileyonlinelibrary.com] *Source*: Reproduced from Chidambaram and Burns 2024.^11^

Nursing homes are unpopular for good reason, and the Trump administration's recent rule changes only exacerbate the problem. Though there have been improvements since the 1980s, quality of care issues remain endemic in long‐term care facilities. A 2022 National Academy of Sciences Report laid out the problems, ranging from insufficient staffing levels and poor infection control to patient neglect and abuse. As one family member commented, “I honestly cannot express how it is. You have to experience it yourself. Being a resident with no dignity, no privacy, no connection to staff, being told what time to eat and shower, at the mercy of staff.”^19^ As Figure 2 shows, hours of care received in nursing homes have been declining over time.^11^ And recent federal rule changes, which would have increased staff levels, were just overturned by the Trump administration.

The bias toward institutions is driven by Medicaid's funding model. Medicaid includes both entitlement and non‐entitlement programs. If you are eligible for Medicaid entitlement programs, such as most children and parent beneficiaries, you receive benefits if you meet the eligibility criteria. Non‐entitlement programs, which are called Medicaid “waiver” programs, work differently. They have fixed budgets or enrollment capacity. Even if you qualify, you may not receive benefits.

The institutional bias results from the fact that institutional care is funded through the entitlement program, whereas the majority of Medicaid home and community programs are funded via waiver, or non‐entitlement programs.^5^ While two‐thirds of Medicaid long‐term care beneficiaries receive home and community‐based services, institutional care remains the only guaranteed benefit. Current estimates are that around 700,000 individuals are on waitlists to receive Medicaid home and community‐based services, though there is significant variation around the country, with states such as Texas, Florida, and North Carolina having especially long waitlists.^5^, ^20^ Although states haven't been required to systematically report how long people wait, it's estimated that disabled people spend an average of three years on waitlists before accessing services.^20^

There is significant concern that waitlists, and access to home and community‐based services (HCBS), will be hit hard by HR1. States are facing what will total nearly $1 trillion in federal spending cuts. States cover about one‐third of Medicaid spending, and it comprises about 30% of state budgets.^21^, ^22^ Unlike the federal government, states can't accrue deficits. Medicaid waiver programs, which cover many home care services, will be the easiest cuts to make. Waivers are optional; states can either cut them altogether or limit how much they spend. It is much easier to cut costs with waiver programs than with any changes to the entitlement program.^23^ Idaho, for example, already has begun cutting therapies for disabled Medicaid beneficiaries, and the governor is proposing even larger cuts to Medicaid disability programs.^24^

### Medicaid Is Difficult for People to Navigate

It's not simply that eligibility criteria are stringent, or that access is not guaranteed for the eligible, the complicated web of different Medicaid programs makes the system incredibly difficult for disabled people, and their caregivers, to navigate.^5^ Indeed, the very people most likely to qualify for these benefits would be largely unable to traverse this process independently.

Federalism ensures that there is significant state variation in how Medicaid programs are designed, administered, and experienced on the ground by the public.^5^, ^25^ Medicaid eligibility processes, including annual requirements to “recertify” eligibility, are notoriously onerous and lead millions to lose coverage due to bureaucratic mishaps.^26^

This baseline complexity is exacerbated for those needing long‐term care because HCBS are largely funded through waiver programs. States choose whether, and how, to fund HCBS. With federal approval, they decide who will benefit, what care will be covered, and how to administer it. Most states have a range of different programs that cover different populations and services. The result is that there are nearly 300 long‐term care waiver programs, and little uniformity in their design and eligibility processes. This opaqueness and complexity make it difficult for the public to sort out what programs and services are available, how to apply for them, and how to navigate complicated administrative requirements.

In practice, the process is opaque, complicated, and incredibly time‐consuming, especially for people with significant disabilities.^27^ It requires understanding complex eligibility rules, gathering extensive documentation, locating the Medicaid office, completing the application, undergoing in‐person disability evaluations by caseworkers or medical personnel, and waiting up to 90 days—or often longer—for a decision. Errors in determination letters are frequent, making the process even more challenging. Up to 35% of these letters contain errors made by Medicaid staff.^28^ Given that the policy is targeted at people with significant disabilities, the degree of complexity is particularly burdensome.

For those wanting HCBS, the complicated eligibility process is just the start. As already noted, in many states, even if an individual completes these processes and is determined eligible, they are often put on a waitlist for HCBS until an open slot or resources become available to fund them. The amount of time lost is often significant, with individuals waiting up to 17 years in some states to access services.^5^ Moreover, states typically provide little information for people on waitlists. For example, people typically don't know how long they will need to wait.

## What Do We Have to Be Optimistic About?

Although the state of long‐term care in the United States is troubled, there are glimmers of hope. Medicaid beneficiaries are far less likely to end up in an institution, and far more likely to stay in their own home, than they were only 30 years ago. Even more promising, the state of Washington made a move to bypass Medicaid altogether—adopting what could eventually be a universal long‐term care insurance program, a critical step away from the current poverty‐based, bureaucratically byzantine system.

### There Has Been Substantial Growth in HCBS

**Figure 3:**
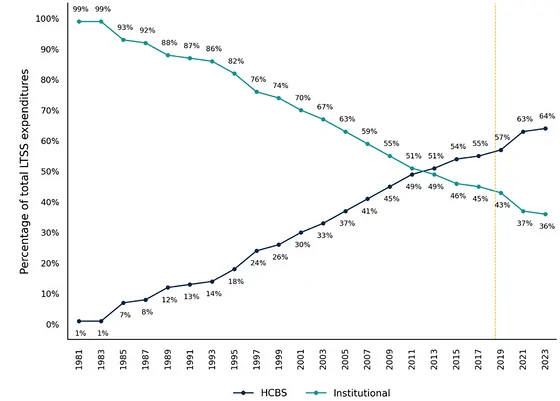
Spending on Home and Community‐Based Services and Institutional Care as a Fraction of overall Medicaid Long‐Term Care Spending [Colour figure can be viewed at wileyonlinelibrary.com] *Source*: Reproduced from Carpenter et al. 2025.^30^

One of the most significant changes to Medicaid is that majority of beneficiaries now receive care in their own homes instead of being forced into an institution. In 1988, Medicaid devoted nearly 90% of its long‐term spending on institutional care. Today it is less than 40%. This has been, in large part, fueled by a move toward funding home care via waiver programs. Non‐entitlement funding, which is nearly all spent on HCBS, has grown from 3% of Medicaid‐funded long‐term care in 1989 to more than 27% today.^29^ As Figure 3 shows, Medicaid spending on HCBS rose from under 10% of total long‐term care spending in the 1980s to 64% by 2023.^30^ Today, more than 70% of people receiving Medicaid long‐term care benefits receive them in their own homes.^15^

There are a few explanations for its growth.^31^ First, community‐based long‐term care is popular, and the public has demanded that disabled people of all ages should be able to remain in the community rather than be forced into institutions.^32^ Public sentiment, and the disability movement, helped generate the legal framework that challenged Medicaid's bias toward institutional care. The 1990 Americans With Disabilities Act (ADA) and the subsequent 1999 *Olmstead v. L.C*. Supreme Court ruling mandated that people with disabilities had a right to live in the community.^33^ Specifically, the Supreme Court ruled that Medicaid was violating the ADA by segregating disabled people within institutions. Although the *Olmstead* decision did not lay out an unconditional right to Medicaid‐funded home care, it did give disabled beneficiaries more leverage to access long‐term care in the community.

### A Move Toward a Universal Long‐Term Care System

While the move away from institutional care, and toward home care, has been a major change, perhaps the most exciting move is the creation of a universal long‐term care social insurance program in Washington State. Many of the problems outlined earlier, from forcing people into poverty to receive care to complicated and onerous eligibility and enrollment processes, are typically not present in universal systems. Moreover, as evidenced by the recent trillion dollars in funding cuts to Medicaid, means‐tested policies typically lack the political resilience of more universal policies like Medicare and Social Security.

Washington is about to stake a new path forward. It will be the first state in the country to offer a universal long‐term care social insurance program. Called Washington Cares, it is funded with a 0.58% tax on wages.^34^ After 10 years of contributions, workers become eligible for a $36,500 lifetime benefit, which will be adjusted for inflation over time. The fund has already accrued $1 billion in its trust fund, with benefits beginning to be paid out in July of 2026.^35^ The only additional eligibility criterion is that individuals need assistance with three or more activities of daily living, such as help with bathing, dressing, or eating.

Will it work? The choice to design a traditional social insurance program, with the program largely mandatory and universal, reflects lessons from prior failures in the United States. The 1988 Medicare Catastrophic Coverage Act, which significantly increased coverage for nursing home care and included robust provisions to prevent spousal impoverishment due to long‐term care costs, was repealed two years later. The financing of the bill, with costs borne solely by Medicare beneficiaries, and thus a move away from a broader social insurance model, ultimately was a key part of its political demise.^36^, ^37^

The next attempt to expand long‐term care coverage was the Community Living Assistance Services and Supports (CLASS) Act, which was part of the 2010 Affordable Care Act, informally named Obamacare. After paying a premium for at least five years and then qualifying as disabled, individuals would be eligible for a daily cash benefit to be used to fund long‐term care support. There were no lifetime benefit limits. CLASS was repealed in 2013 after it became clear the program was fiscally unsustainable, in large part due to adverse selection resulting from voluntary participation.^38^, ^39^ While not requiring participation meant it didn't carry the political risk that undermined the Medicare Catastrophic Coverage Act, it also meant that not enough healthy individuals would enroll to ensure the program's fiscal health.

In contrast to these failed prior efforts in the United States, there has been substantial success in other countries that have employed a social insurance model, where costs are spread across the full population, and benefits are more universal.^40^ Germany, which established its social insurance long‐term care system in 1995, has proved successful, both in terms of financing and political resilience.^41^ Japan established a social insurance model in 2000, which has also largely been successful.^42^ Additional examples include the Netherlands, South Korea, and Luxembourg.^43^ Other countries, such as in Scandinavia, cover long‐term care but through their broader universal health insurance system. Because the United States lacks universal health insurance, this is not a feasible model.

Another design feature of Washington Cares, which reflects successful models of long‐term care provision around the world, is that it ensures flexibility in service provision. Funds can be used to purchase either home‐based or institutional care—a significant shift from the institutional bias still present in Medicaid.^35^

Although very promising, there are uncertainties as the program moves forward (Munnell 2024).^34^ The benefit is relatively small given overall long‐term care costs. The state has partnered with private long‐term care insurance companies to allow individuals to supplement the coverage. Given the relative failure of this market, however, it's not clear whether this will be effective.

The other outstanding question relates to the design processes to determine whether one's disability is qualifying. These processes can be onerous and challenging for people to navigate.^5^ In practice, countries like Germany, for example, have struggled to effectively implement these processes, requiring repeated reforms over time.^44^

The biggest threat to Washington Cares, however, was recently defeated. In the fall of 2024, there was a referendum, promoted and funded by a conservative hedge fund manager, to make the program voluntary.^45^ As evident from the CLASS failure, this would have certainly killed the program. The referendum failed, however, with 56%of Washington residents supporting the compulsory approach to funding.^46^

## Conclusion

The United States’ poverty‐based public long‐term care funding structure means that most older adults must become poor to access services and supports that are otherwise unaffordable, except to the very rich. Nonetheless, there is reason for cautious optimism about its future.

One reason is the ongoing shift in Medicaid funding from institutional to community‐based care, which has improved quality of life for millions of disabled Americans. The roots of the current system lie in the fact that the 1965 Medicare legislation only covered acute care, whereas Medicaid included nursing home care as a mandatory benefit.^47^, ^48^ As late as the early 1980s, 99% of Medicaid long‐term care spending was on nursing care. A series of legislative changes and court challenges, instigated in large part by the disability rights movement, led to a gradual shift, such that today two‐thirds of Medicaid long‐term care funding is provided to the aged and disabled in the community.

Though access to home and community‐based care has grown quickly, the system, in part due to its ad hoc and incremental expansion, remains riddled with problems. The ability to access home care is conditional, funded by voluntary state waivers, and even those able to ultimately navigate the byzantine bureaucracy shaping access may still be left on yearslong waitlists.^5^ Moreover, because these benefits are often not required, there is growing fear that access will shrink as states face difficult budgetary choices due to the nearly $1 trillion cut to federal Medicaid payments from HR1. Indeed, Idaho just enacted cuts to these services.^24^ While the future looks more uncertain than it did a year ago, compared to 30 years ago, the ability to access that care has grown by leaps and bounds.

The most radical potential shift, however, is Washington State's innovative universal long‐term care social insurance program. When Medicare was created in 1965, the choice to make it means‐tested, rather than a social insurance benefit, explains much of the ongoing weaknesses in the system. The experience of other countries demonstrates that broad‐based, compulsory social insurance is effective—lessening poverty‐induced eligibility hurdles; increasing access to care, especially in communities; and mitigating political vulnerability. Ultimately, as the population ages and care needs grow more urgent, the United States faces a critical choice—cling to a piecemeal, confusing, and impoverishing long‐term care system, or embrace universalism to ensure meaningful access to needed community‐based long‐term care supports.
